# Burden of delayed discharge on acute hospital medical wards: A retrospective ecological study in Rome, Italy

**DOI:** 10.1371/journal.pone.0294785

**Published:** 2024-01-24

**Authors:** Antonio Vinci, Giuseppe Furia, Vittoria Cammalleri, Vittoria Colamesta, Patrizia Chierchini, Ornella Corrado, Assunta Mammarella, Fabio Ingravalle, Dorian Bardhi, Rosa Maria Malerba, Edoardo Carnevale, Susanna Gentili, Gianfranco Damiani, Corrado De Vito, Massimo Maurici

**Affiliations:** 1 Hospital Health Management Area, Local Health Authority “ASL Roma 1”, Rome, Italy; 2 Doctoral School of Nursing Sciences and Public Health, University of Rome “Tor Vergata”, Rome, Italy; 3 Department of Public Health and Infectious Disease, Sapienza University of Rome, Rome, Italy; 4 Hospital Health Management Area, Local Health Authority “ASL Roma 6”, Albano Laziale, Italy; 5 Post-Graduate School of Hygiene and Preventive Medicine, University of L’Aquila, L’Aquila, Italy; 6 School of Specialization in Hygiene and Public Health, University of Rome “Tor Vergata”, Rome, Italy; 7 Aging Research Center, Department of Neurobiology, Care Sciences and Society, Karolinska Institutet and Stockholm University, Stockholm, Sweden; 8 Department of Health Sciences and Public Health, Section of Hygiene, Catholic University of the Sacred Heart, Rome, Italy; 9 Department of Biomedicine and Prevention, University of Rome “Tor Vergata”, Rome, Italy; San Giuseppe Hospital, ITALY

## Abstract

**Introduction:**

Delayed discharge represents the difficulty in proceeding with discharge of patients who do not have any further benefit from prolonged stay. A quota of this problem is related to organizational issues. In the Lazio region in Italy, a macro service re-organization in on the way, with a network of hospital and territorial centers engaged in structuring in- and out- of hospital patient pathways, with a special focus on intermediate care structures. Purpose of this study is to quantify the burden of delayed discharge on a single hospital structure, in order to estimate costs and occurrence of potential resource misplacement.

**Material and methods:**

Observational Retrospective study conducted at the Santo Spirito Hospital in Rome, Italy. Observation period ranged from 1/09/2022, when the local database was instituted, to 1/03/2023 (6 months). Data from admissions records was anonymously collected. Data linkage with administrative local hospital database was performed in order to identify the date a discharge request was fired for each admission. Surgical discharges and Intensive Care Unit (ICU) discharges were excluded from this study. A Poisson hierarchical regression model was employed to investigate for the role of ward, Severity of Disease (SoD) and Risk of Mortality (RoM) on elongation of discharge time.

**Results:**

1222 medical ward admissions were recorded in the timeframe. 16% of them were considered as subject to potentially elongated stay, and a mean Delay in discharge of 6.3 days (SD 7.9) was observed.

**Discussion and conclusions:**

Delayed discharge may cause a “bottleneck” in admissions and result in overcrowded Emergency Department, overall poor performance, and increase in overall costs. A consisted proportion of available beds can get inappropriately occupied, and this inflates both direct and indirect costs. Clinical conditions on admission are not a good predictor of delay in discharge, and the root causes of this phenomenon likely lie in organizational issues (on structure\system level) and social issues (on patient’s level).

## Introduction

### The problem of delayed discharge

Delayed Discharge represents the difficulty, in hospital ordinary wards, in proceeding with discharge of patients that are still admitted, but do not have any further benefit from prolonged stay [1, 2]. This usually happens after situations with resolution of the acute event that brought the patient to the hospital, but with the persistence of other problems that stall discharge, and do not always have a clinical root cause. A fundamental role in this is often played by social and organizational components [3].

Among organizational components, we can list the waiting time for bed availability within a structure of lesser intensity of care, or waiting times due to territorial services activation. Among social components, most impact comes from the patient’s frailty condition, with elements (such as comorbidities, dementia or difficulty in performing normal daily activities, not to mention psychiatric or social disabilities and\or disadvantages) that are known risk factor for increase length of stay in hospital ward [4–6]. Also, the very same frailty condition is known to be a predictor of increased and inappropriate use of health services [7]. Social factors are present in a large percentage of emergency department (ED) frequent user group: loneliness, poverty, poor quality of life, difficulties in daily self-management [8–11].

Delayed discharge has been subject of many studies, and it is believed to have a significant impact on both direct and indirect costs. Among direct costs we can find primary admission costs, and secondary costs due to complication during the excessive hospitalization time [12]. Among indirect costs, we can find those relative to the missing service provision due to the unavailability of a bed occupied by a patient who has already been treated, without further clinical necessities. Also, indirect costs are those relative to the inefficient usage of ward personnel, that is not used at its best in order to solve acute clinical problems, or problems that deserve an hospital admission [13]. Adding on to this, the experience of delayed discharge has a strong impact on the patient, who has to deal with anxiety elements due to feeling rejected and “in the wrong place”, and on assistance personnel, who is often unsatisfied with the whole discharge process [14–16].

Moreover, ethical themes, relative to the increasing inequality towards frailty patients in relation of their usage of health services that are on paper available to them, increase the complexity of this matter. Actually, it is well-known that frailty patients who undergo delayed discharge are exposed to a clinical treatment inferior in quality, even in the rehabilitation setting, causing an increased risk of functional decline, falls, and adverse events such as Healthcare Associated Infections, complications, and errors in therapy [17–20].

Last but not the least, delayed discharge has a heavy impact on the appropriateness of setting of care, as it hinders the opportunities of making good use of hospital resources and anticipating clinical readiness for discharge [21]. This inflates overall management costs, with a bottleneck phenomenon given by the unavailability of hospital beds, especially in hospital with active ED [3, 22–25].

Several proposals have been made, in the hope of keeping in check the occurrence of delayed discharge. Most initiatives have been implemented on hospital levels, rather than in a more comprehensive way [26–30].

It has been observed that the phenomenon is related to expenditure cuts in social expenditure, and interventions towards the increase of the beds availability in intermediate care structures are suggested by many authors [31, 32].

Likewise, it would be opportune to offer frailty people a wide range of possibilities for their subsequent treatment after an acute event, in order to promote their independence after their admission. For this reason, interventions with a strong coordinated component of social and health services, be it institutional, private or volunteer (i.e. charities), have an high impact on the matter [33].

Beyond macro scale strategical solutions, further ideas have been proposed for implementation on micro scale, including strict monitoring of patients with high admission duration, initiatives towards bettering communication among clinicians and families, and models in order to ensure accountability of personnel on the subject [26, 34, 35]. Active patient involvement is also considered an important aspect; however, in the Italian context, research on patient involvement in the caring process is in its infancy, and the existing studies highlight poor engagement in this process by healthcare professionals [36].

In Italy, in most recent years, there has been a continuous reduction in beds availability, due to spending cuts and budget reasons, on a regular basis and on nation-wide level. This was very impactful during COVID-19 emergency, as the health system was put under strong pressure, due to reductions in hospital beds and capacity and underinvestment in community-based care [37]. This meant that it was necessary to increase beds usage efficiency, dealing with healthcare demands despite a reduction in available resources.

In such scenario, evaluating and quantifying the problem of delayed discharge deserve a specific and deep attention, especially at the hospital level, being the operative element of the healthcare system most directly involved in this process.

### Patient discharge process in the Italian National Health Service

**Fig 1:**
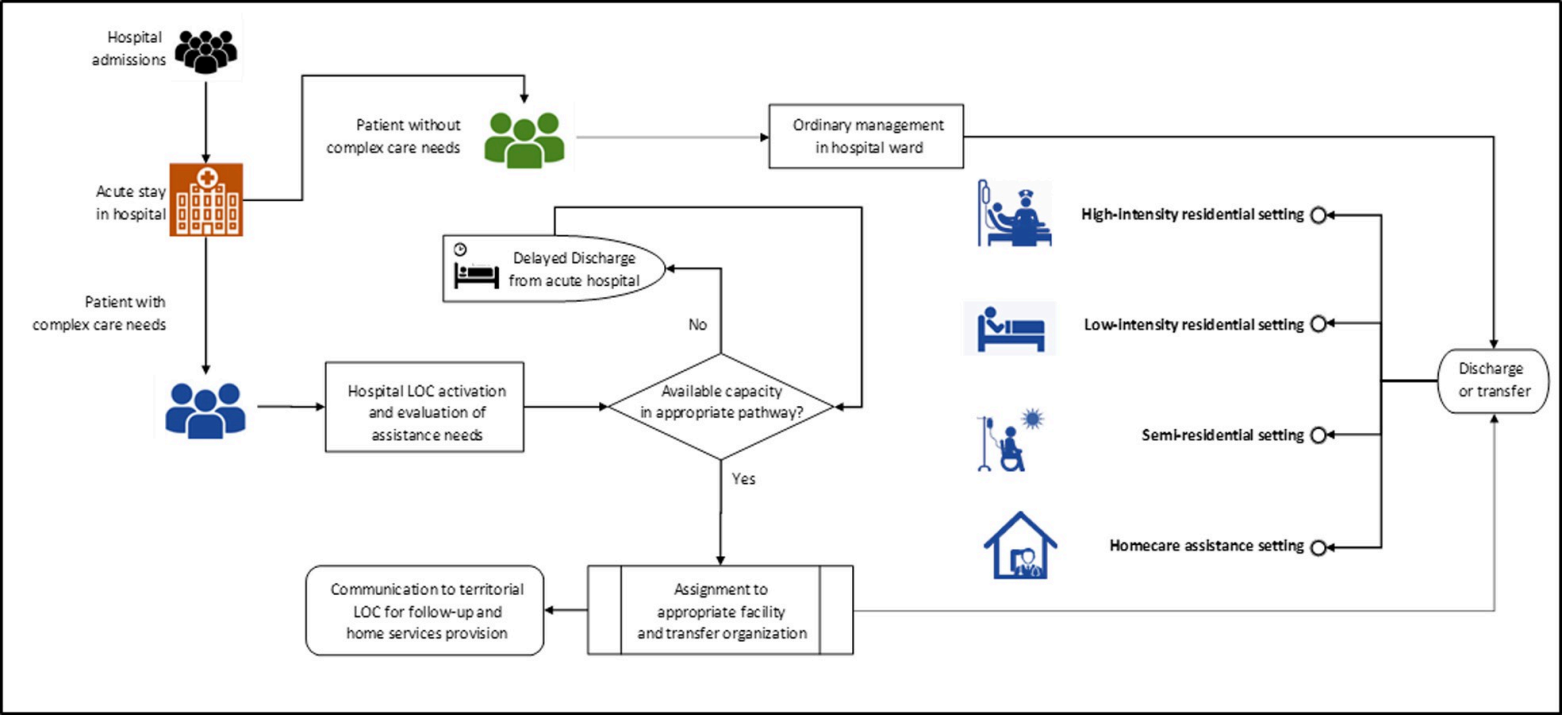
Organization of intermediate care services in the Italian National Health Service (NHS). Adapted from *Onen-Dumlu et Al*. with permission from the Authors [38].

In the Italian National Health Service (NHS), intermediate care facilities fill the gap between acute hospital and usual place of residence. The rationale is that patients are admitted into acute hospital and treated for their acute illness, and then dismissed either home, or in a territorial structure that is better suited for their needs, as soon as there is no further need of acute care. This model is detailed in **Fig 1**, and is widely shared in European and American Health systems [38]. Italian NHS is a Beveridge health system which has adopted DRG payments to hospitals; Italian DRGs follow the US (CMS) model. In order to obtain a DRG payment, each structure must generate and transmit Hospital Discharge Forms (*SDO—Scheda Dimissione Ospedaliera*), in anonymous form, for every admitted patient [39].

Italian health system provides not only for hospital assistance, but also for intermediate, homecare, and territorial (outpatient and diagnostics) assistance. In 2007, the national government produced documentation on the residential and semi-residential typologies of assistance, distinguishing between Social-oriented and Health-oriented [40].

The main assistance structures can fall into one of the following categories:

- Care for elderly and frailty people;
- Rehabilitation facilities;
- Care for disabled people;
- Psychiatric care;
- Palliative care.

These structures can operate on a full residential regime (full 24h/die), on a semi-residential regime (patients access on daytime and goes back home on nighttime), or on a home-care basis. Some structures can take care of patients in different regimes at the same times [41]. **Table 1** synthetizes the different typology of structure provided by NHS, either directly or indirectly via convention and accreditation programs.

**Table 1:** Non-acute care facilities provided by Italian NHS.

Assistance level	Description
**Health facilities for Elderly people**	
R1	Patients needed intensive treatment, such as invasive mechanical ventilation, or other support to vital functions.
R2	Non self-sufficient patients with healthcare needs, such as daily nursing or medical care.
R2D	Patients with senile dementia\Alzheimer disease, with need of rehabilitation, re-orientation or personal tutelage, with need of both daycare and night care.
R3	Non self-sufficient patients with long-term assistance needs, with low need of healthcare assistance.
SR	Semi-residential maintenance assistance.
SRD	Semi-residential assistance for patients with senile dementia\Alzheimer disease, with need of rehabilitation, re-orientation or personal tutelage but no need of night care.
**Extensive rehabilitation facilities**	
RRE1	Post-Acute Extensive Rehabilitation for care prosecution after intensive care, or for patients who cannot undergo intensive treatment.
RRE2	Long-term Post-Acute Extensive Rehabilitation for treatment against chronic, evolving and invalidating illnesses.
**Social and Health care facilities for Disabled people**	
RD1	Diagnosis, therapy and rehabilitation for disabled people with need for extensive or intensive rehabilitation, and maintenance treatment for patients in need of high intensity level of care, including minimally responding patients.
RD2	Diagnosis, rehabilitation and therapy for children with behavior disturbs, or neuro-psychiatric syndromes.
RD3	Maintenance therapy and rehabilitation, coupled with tutelage for people with strong disabilities.
RD4	Maintenance therapy and rehabilitation, coupled with tutelage for people with strong disabilities and no family support.
**Facilities for people with Psychiatric needs**	
RP1	Care and rehabilitation post-acute programs. Treatment duration and precise therapeutic objectives must be defined before admission.
RP2	Facilities for people pa who are partially self-sufficient, but cannot be assisted in their own family environment and need housing, with some healthcare tutelage.
SRP	Semi-residential facilities for patients with psychiatric needs and no need of night care.
**Facilities for Palliative care**	
H1	Residential palliative care
H2	Homecare palliative care
HP	Pediatric palliative care

### Local background

Local Health Authority (ASL) Roma 1 is the Public Health Authority in charge of the Historical Centre and North-western area of the Metropolitan city of Rome, Italy. It has a resident population of over 1 million, extending on an area of 524,0 km^2^, which is almost 40% of the metropolitan city of Rome [42]. Territorial needs are managed through 6 district, with General Practitioners, outpatient clinics and lab facilities. In terms of ordinary hospital wards, ASL Roma 1 directly manages two hospitals, St. Spirito (SSP) and St. Filippo Neri (SFN), and it commits services towards 11 other private hospitals in the area, in accreditation regime [43].

**Fig 2:**
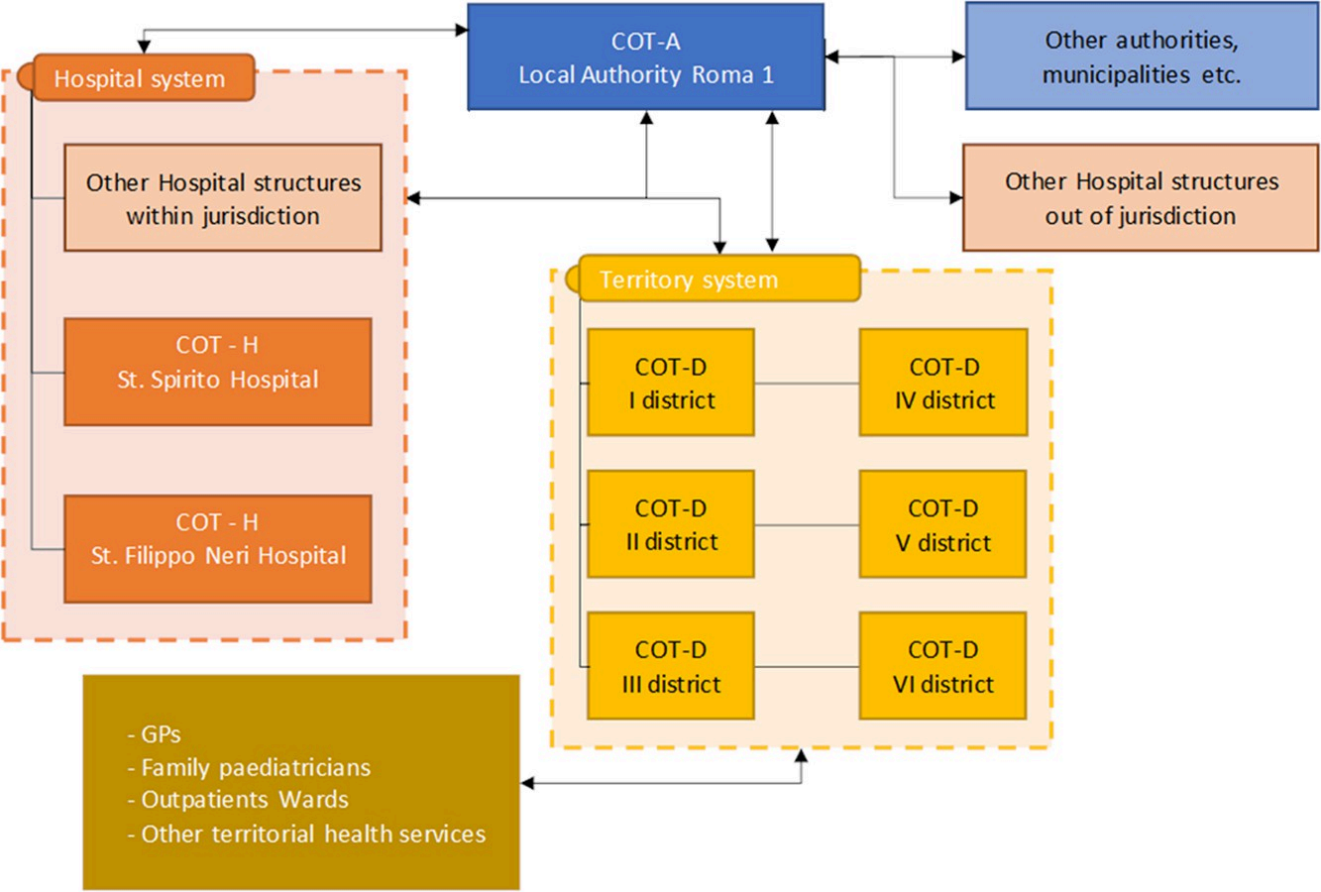
Roma 1 operation centers model for discharge management. COT-A: Authority Center of Operation; COT-H: Hospital Center of Operation; COT-D: District Center of Operation; GP: General Practitioner.

In order to facilitate patients’ flow across the wards of SSP and SFN, especially for patients with complex needs who cannot be directly discharged home, Roma 1 adopted a network model, in which every directly managed structure has its own Center of Operations (COT), staffed with both social and healthcare personnel, communicating with a Central Hub and with external territorial structures. This is structured in order to allow clean management of patients’ flow across each service, and is built up in order to better territorial-hospital integration across the area (**Fig 2**).

Hospital Clinicians had the option to request COT support in case they had difficulties with discharge process, either bureaucratic, or depending from complex social situations or in need of specific evaluation (such as the case for palliative care). SSP hospital required COT intervention for all patients in need of nursing home care or admission to secondary care structure; SFN hospital allowed instead full discretion to the treating physician when deciding if involving or not COT in patient management.

This model started initially in small scale, and became fully operational at the end of September 2022. Each COT was provided with a specific data analysis tool that was internally developed and used for case management and for general reporting to Management Office.

### Study objectives

Primary objective of present study is to quantify the impact of delayed discharge on a hospital ward bed availability, both in terms of inappropriate bed usage, and in terms of economic direct costs.

Secondary objectives are to understand the most common reasons behind the Delayed Discharge phenomenon, assuming that the causes are likely to lie in potential unknown or unmet needs of the impatient population, especially given the severity of the affliction that led them to hospital ward admission in the first place, and to investigate characteristics of patients deceased while awaiting discharge.

## Materials and methods

### Study design

This is an Observational Retrospective study; Ethical Board approval n° 238/CE Lazio 1. Patient Consent was waived because the study was conducted on data routinely collected for administrative purpose. Data was fully anonymized before being accessed for analysis.

### Setting and participants

All patients admitted in SSP Hospital in ordinary medical (i.e. non-surgical) wards, for which was requested social\healthcare support in discharge to the COT, and who were either discharged or deceased during the process, were eligible for inclusion in the study. Patients admitted in Intensive Care Unit (ICU) wards and discharged to other acute or intermediate care facilities were not included, since they represent a peculiar subgroup of patients with peculiar needs [44]. However, patients admitted to ICU, transferred to a different in-hospital ward and then discharged were eligible for inclusion. The involved medical wards were Cardiology, Internal medicine, and Emergency medicine, as no other medical ward is active in SSP hospital beside those mentioned.

Patients who had no problematic delaying their discharge, thus not being referred to COT, were not eligible for inclusion neither those admitted in non-ordinary regimen (day hospital, day surgery or outpatient setting). Gynecology, neonatology and obstetric wards were excluded from this analysis as well, since they represent the needs of a very specific set of patients, who mostly do not need COT-H activation anyway. **Table 2** details the characteristics of the wards included in this study.

**Table 2:** Characteristics of SSP hospital wards included in the study.

Ward type	Beds availability	Admissions	Patients’ LOS (days)	COT-H assisted discharges	eLOS in COT-H assisted discharges (days)
**Internal Medicine**	25	245 (primary) + 416 (secondary)	14.9	188 (31 deceased)	7.3
**Emergency Medicine**	13	178	5.9	76 (11 deceased)	4.6
**Cardiology**	17	383	5.1	22 (3 deceased)	3.4
**Total Medical Area**	**55**	**1222**	**7.9**	**286 (45 deceased)**	**6.3**

Beds availability defined as average of active beds during the investigation period (rounded without decimals). LOS: average Length of Stay during the investigation period. Patients discharged to homecare are included in this count.

In order to better depict Wards activity, the Barber nomogram was used. Barber nomogram allows the setup of a range of values in order to assess the performance in the delivery of care [45].

Observation time ranged from 1 September, 2022 to 1 March, 2023. Data was collected on 1 March, 2023.

### Variables and data sources

Principal investigators (AV and GF) had full access to each COT hospital database, due to the respective Operating Role within the Local Health Authority. All data were extracted anonymously and put into a comprehensive dataset that was subsequently used for statistical analysis and reporting. In this phase, data was cleaned by hand, correcting misspells and obvious inaccuracies (such as wrong admission or discharge century).

Two investigators (AM and OC) had access to SDO database, used for calculation of All Patient Refined-Diagnosis Related Group (APR-DRG, 3M Company, Pioltello, Italy) APR-DRG is an instrument to assess illness severity and defined as risk for in-hospital death (on a scale ranging from 1 to 4 for both items) [46].

Linkage between SDO repository and COT databases was done via the unique admission identifier.

No other database was used for this study.

The following variables were retrieved:

- From SDO repository;

 ⚬ ICD-9-CM diagnosis code.
 ⚬ Aggregate hospital wards volume data:
    ▪ Number of total admitted patients.
    ▪ Number of total discharged patients.
    ▪ Average Length of Stay (LOS).
    ▪ Number of total days of stay.

- From COT database:

 ⚬ Patient admission identifier.
 ⚬ Hospital.
 ⚬ Discharge ward.
 ⚬ Date of potential discharge.
 ⚬ Date of actual discharge.
 ⚬ Discharge type.

“Potential discharge” was defined as the day the patient had no more need of acute hospital care, and was requested for specific intermediate care assistance.

Since all investigated data were required for the admission\discharge process, there was no missing data.

### Sources of Bias and Bias reduction

A potential source of Bias has been recognized in patients that experience a potential delayed discharge are those who are the most frailty and with a more severe illness. In order to quantify this, the All-Patient Refined Diagnosis-Related Groups (APR-DRG) Severity of Illness (SOI) and Risk of Mortality (ROM) was calculated and used for secondary and sensitivity analyses. These scores have already been proven to be useful for hospital or benchmarking and epidemiological analysis, and also to define economic burden and overall DRG value [47].

### Statistical methods

Data were summarized using absolute and relative frequencies for categorical variables, mean, median and range for numerical variables. Chi-squared (*χ*^2^) test for association was used to investigate the relation between categorical variables. Patients were subdivided based on the admission ward, and on the post-discharge facility they were programmed for, in order to better understand and quantify the needs of this specific population.

In order to control for confounding, a separate analysis on SOI and ROM was performed, considering ROM as a potential moderator variable of SOI, and evaluating their impact on discharge delay. In this form of sensitivity analysis, null hypothesis (*H*0) was that SOI and ROM do not influence inappropriate excessive LOS (eLOS).

Given the investigated setting, a hierarchical Poisson regression model (Patient < Ward) was proposed for inferential analysis. This model was used because eLOS had a Poisson distribution rather than a normal one. SOI and ROM were tested for correlation using Pearson’s ρ, while their distribution was investigated using Wilcoxon signed-rank test. ROM role was then investigated as SOI moderator variable.

Wards were considered as the superior grouping level. Then, a similar model but with discharge type as predictor instead of wards was investigated.

Italian Ministry for Economy and Finance provided an estimation of €670 as the daily cost of an acute hospital bed occupation; this was used for a quick estimation of direct costs of delayed discharge [48].

The REporting of studies Conducted using Observational Routinely-collected Data (RECORD) guidelines were used for reporting [49].

## Results

### Participants

2618 patients (1097 M, 1521 F) were admitted in the SSP hospital from 01/09/2022 to 01/03/2023. Of those, 806 (30.7%) were admitted directly in a Medical Ward, while other 416 (15.9%) were admitted in the Internal Medicine Ward as secondary admission, after a primary Surgical/ICU admission. Thus, the patients admitted in the SSP Medical Wards were 1222 (46.7%). Among those, for 186 (15%) COT-H assistance was requested due to intermediate care facility needs, while 81 (6.6%) were in need of homecare or social support. The remaining 955 (78.2%) patients were discharged without necessity of COT-H activation.

**Fig 3:**
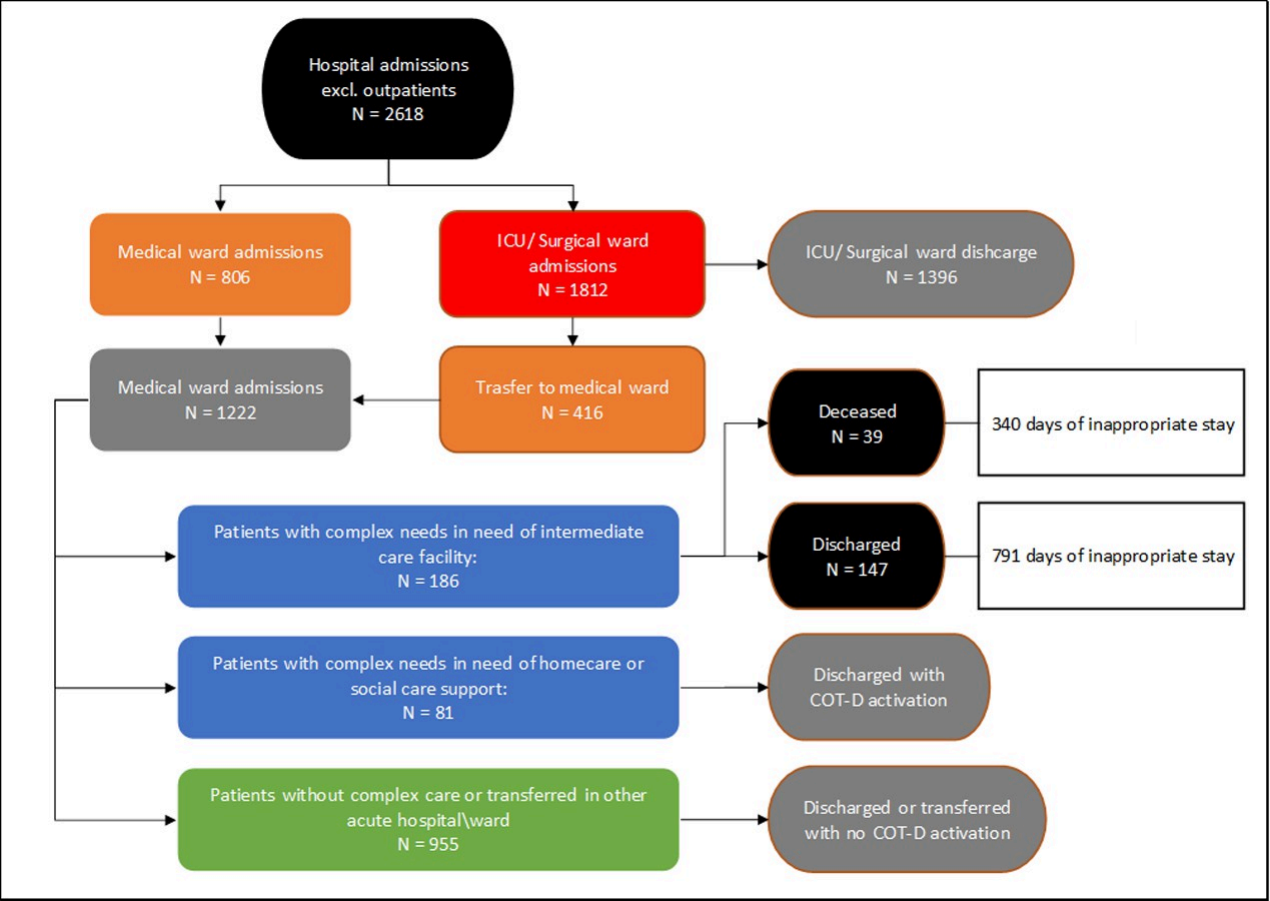
Study patients flow. COT-H: Hospital Center of Operation; COT-D: District Center of Operation.

**Fig 4:**
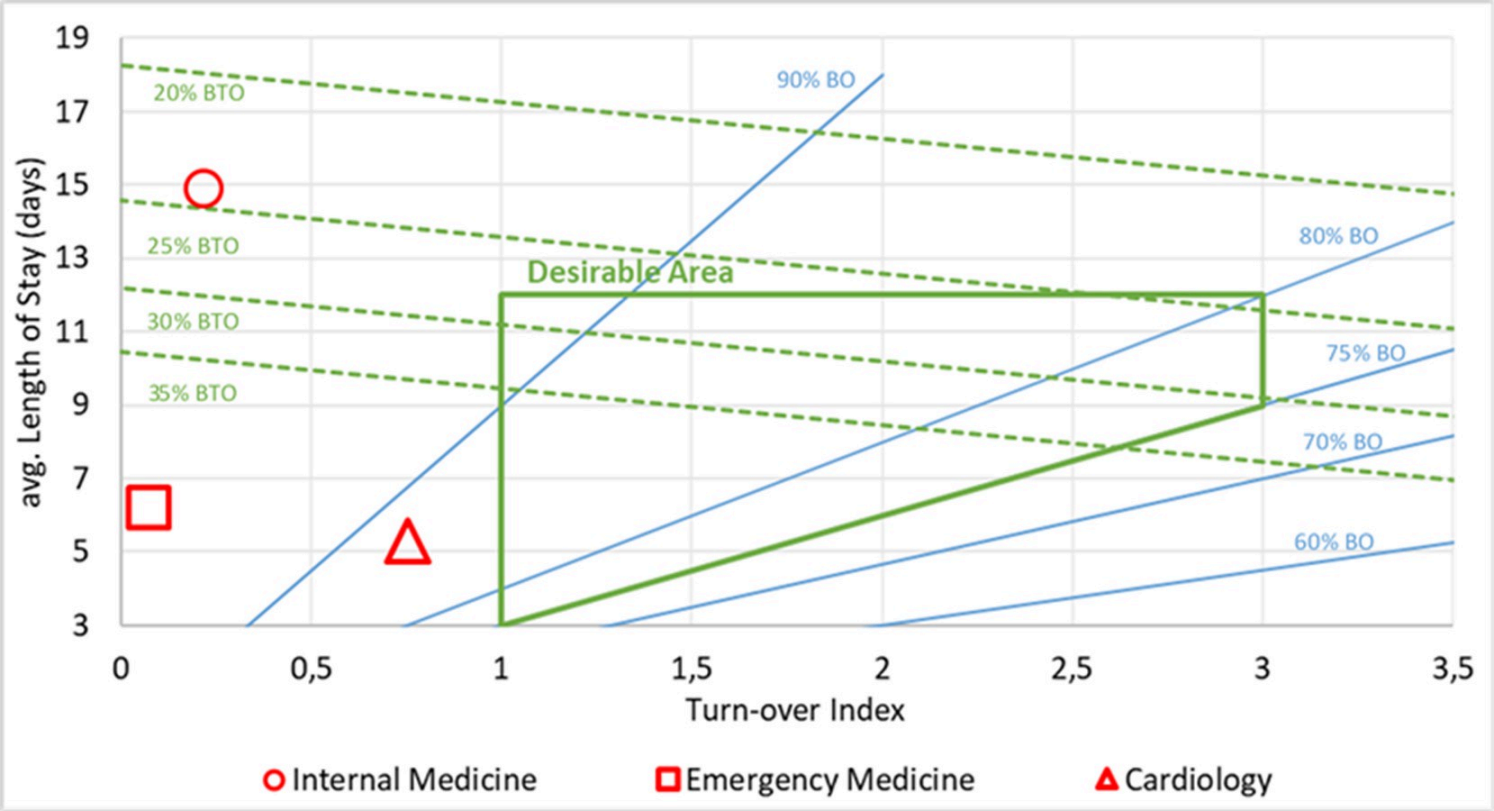
Barber nomogram for the wards included in the study in the selected period. Dashed green lines represent Bed Turn-Over percentage (BTO). Solid Blue lines represent Bed Occupancy percentage (BO). Solid Green Area represent the desirable area for medical divisions.

Distribution of patients among wards is synthetized in **Table 2**. The patients’ study flow is depicted in **Figs 3** and **4** depicts each ward occupation status during the mentioned period using a Barber nomogram; its implications are described in the **Interpretation** section.

### Main results

During a 6-months observation period, a consistent proportion (nearly 22%) of medical patients has been in need of some form of intermediate care assistance from Italian NHS. 103 patients (36%) were in need of RRE1. These represented the largest group, followed by Nursing Homecare (68, 24%) and Palliative Care (64, 23%). A more detailed description is provided in **Table 3**, where LOS of these patients are also provided.

**Table 3:** Raw estimation of yearly cost of inappropriate hospital beds’ occupation.

Ward type	Patients with delayed discharge (N)	Mean delay of discharge in days (SD)	Cumulative Length of delayed assistance (days)	Mean beds used for delayed discharge (% of ward beds)	Yearly costs of Delayed Discharge (€)
**Internal Medicine**	177	7.4 (8.6)	1306	8 (32%)	1,750,040.00
**Emergency Medicine**	73	4.7 (6.1)	332	2 (8%)	444,880.00
**Cardiology**	20	3.5 (2.7)	72	1 (6%)	96,480.00
**Total Medical Area**	**270**	**6.3 (7.9)**	**1710**	**11 (20%)**	**2,291,400.00**

A separate analysis of time spent while awaiting available bed in discharge destination shows that patients in need of RRE1 facilities are the ones most impactful on all wards, with a mean delay from discharge of 7.5 days (SD: 8.7).

### Secondary results

SOI and ROM were found to be strongly correlated (Pearson’s ρ = 0.77), while no significant difference in their distribution was found using Wilcoxon rank-signed test (p = 0.15). No significant difference has been found in the patients’ distribution between admitted wards and discharge facility (Table 4). Likewise, no significant difference has been found in LOS distribution by ward nor by discharge facility. Likewise, sensitivity analysis done using a Hierarchical Poisson regression model (Patient < Ward) between eLOS, SOI and ROM did not show any association significance, with the exception of R3 discharge and low SOI\ROM; this is of course expected because patients discharged to such facilities are typically patients with no further need of medical assistance. On the other hand, the facility type has a much stronger impact on eLOS, with highest delay observed for low-level care assistance needs (mean impact of almost 20 days), regardless of SOI\ROD moderation effects.

**Table 4:** Distribution of patients by wards and discharge facility.

Ward	Hospice	Other	R3	RRE1	Total
**Emergency Medicine**	12	2	6	27	**48**
**Internal Medicine**	49	3	5	66	**123**
**Cardiology**	3	1	2	10	**16**
**Total**	**64**	**6**	**13**	**103**	**186**

Patients discharged with public-assisted nursing homecare were excluded.

Nested Poisson regression model results are provided in Table 5.

**Table 5:** Poisson regression model results and marginal effect of facility on exceeding length of stay.

Predictor variable	Coefficient	95% CI	p-value
**SOI (ref: 0)**			
**1**	0.48	-.031–0.99	0.07
**2**	0.67	0.2–1.15	0.01
**3**	0.58	0.15–1.02	0.01
**4**	0.40	-0.05–0.85	0.08
**ROM (ref: 0)**			
**1**	-0.22	-0.54–0.10	0.18
**2**	-0.47	-0.72 –-0.22	0.01
**3**	-0.43	-0.64 –-0.22	0.01
**4**	N\A	N\A	N\A
**Discharge facility (ref: homecare)**			
**Other**	0.48	0.19–0.77	0.01
**RRE1**	0.35	0.19–0.51	0.01
**R3**	1.05	0.74–0.22	0.01
**Hospice**	-0.36	-0.85–0.13	0.15
**Ward (ref: emergency medicine)**			
**Internal Medicine**	0.31	0.04–2.47	0.01
**Cardiology**	0.01*	0.01–0.01	0.01
**Marginal predicted mean effect (days)**		**95% CI**	**p-value**
**Homecare**	6.8	3.1–10.5	0.01
**Other**	10.9	4.4–17.5	0.01
**RRE1**	9.6	4.4–14.9	0.01
**R3**	19.3	7.6–30.9	0.01
**Hospice**	4.7	1.4–8.1	0.01

Effect of predictors on extended Length of Stay (days). SOI: Severity of Illness. ROM: Risk of Mortality. Ward is in the model a second-level predictor variable.

## Discussion and conclusions

### Key results

Patients admitted in SSP hospital medical wards who were recognized as a “difficult discharge” were needlessly prolonging their acute stay of almost a week (Mean delay in discharge value 6.7 days, SD 7.9 days). Almost one fifth of SSP patients occupying a bed at any given time in a Medical ward is likely being treated in an acute setting in an inappropriate way. Prevalence of Delayed discharge was found to be in line with current literature, albeit unequally present, and too high in some wards compared to others (with values ranging from 6% to 32%). As comparison, a recent meta-analysis of Landeiro et al. found it ranging from 1.6% to 91.3%, depending on the context, with an average of 22.8% [50]. SOI and ROM do not play any role in determining the prolonged LOS, since they become an independent variable once a patient is fully treated and ready for discharge. This means that delay in discharge is due to systemic and organizational causes, such as demand and supply of intermediate and homecare facilities, rather than clinical reasons.

### Limitations

Data used for this study were created for administrative purposes. This limitation was however overcome by the possibility of performing data linkage with local clinical digital platforms, so that the resulting summaries did encompass the entire discharged population. This allowed the usage of descriptive statistical framework in most of the analysis and reporting, with inferential issues kept to a minimum.

Usage of a standardized tool for SOI and ROM (APR-DRG tool) increases the quality of evidence of the study, as it allowed sensitivity analysis and the clarification of potential confounding factors [47].

The final result is a downward estimate of the real necessity, since it monitored only publicly-provided assistance: it is possible that patients may have chosen to get discharged, and then provide for intermediate out-of-pocket assistance by themselves. While this is unlikely for the case of residential acute care and palliative care, it is a possibility in case of low-intensity homecare, for which unprofessional caretakers are often employed in Italy, sometimes partaking an informal economic network whose extent is hardly tracked [51]. Such situation is common in other European countries, although organizational differences and state funding or expense participation may vary [52].

Lastly, a potential limitation may lie in the un-adjustment for COVID-19 related issues. However, there is no evidence to suggest that COVID-19 had any impact on the investigated phenomenon. While it is true that COVID-19 had a great impact on health system worldwide, this was mostly true in its early stages and on emergency departments, in both hospital wards and pre-hospital services [53–58].

On the other hand, delay of clinical discharge should be independent from the patients’ conditions. Destination facilities may require some further safety procedures (and consequent bureaucratic delay) in case of COVID-19 infection, but while this had a substantial impact in the early pandemic stage (2020–2021), after the full-fledged Italian vaccination campaign and the subsequent lifting of many restriction measures COVID-19 was fairly manageable from the organizational standpoint; thus it should have had little to no impact in affecting present study conclusions [59–63]. Evolving population knowledge, attitude, and perception of vaccination effects also played a role, as positive intention in vaccination meant a higher vaccine coverage in the following months, and therefore impacted on COVID management [64, 65]. As is known, vaccine hesitancy played an important role in COVID, even more than what is expected for other type of pathogens; such behavior impacts on disease spread and consequent drug prescription attitudes on the territory, and this all has a foreseeable impact on hospital demand [66–68]. In particular, antibiotic over prescription (by all types of health personnel) is constantly been linked with the occurrence of antimicrobial resistance, and thus with more difficulties in patient care management and further hospitalization increase [69–72].

During the study period, a positive swab, regardless of symptoms, forced clinicians to isolate the patients, thus reducing the number of available beds, also in the intermediate facilities. This may have had an impact on the length of stay of patients, due to reduced output supply. As a matter of fact, a high delay is found for low-level assistance facilities, while homecare solution are superseded only by palliative care provisioning. It must be noted however that such provisioning can be provided in both residential and homecare setting. Since home-based solution have already been proved the most optimal from both clinical and logistical point-of-view, a more proactive use of such setting is to be expected, even using novel approaches such as telemedicine and IT-based solutions [73–75].

### Interpretation

The main results of this study show that, in SSP hospital, up to 20% of available hospital beds were occupied by patients who could have had more benefit in a different setting, and were also subtracting the ward bed to other patients awaiting for hospital admission from ED, potentially resulting in chronic ED overcrowding. This situation appears to be so common worldwide that is often taken as a given among health practitioners, even in literature [76–79]. It is also responsible of staff stress and burnout, as result of constant high bed occupancy despite high bed turnover and low ward LOS [13]. This situation was indeed present in SSP hospital, as Fig 4 shows: while there was an efficient usage of beds, LOS was above average and admissions were difficult. Moreover, high-level specialty wards are usually encouraged to transfer their patients to generalist wards in order to free specialty beds, henceforth dragging down their own average LOS while not influencing patients’ overall LOS. This behavior can be further reinforced by opportunistic or poorly designed incentive mechanisms (such as budget increments in case of ward LOS under a certain threshold). On the other hand, delay in discharge process represent an important cost for the structures experiencing it: for SSP, it resulted in a yearly loss of over 17% of total medical wards production value, due to assistance in inappropriate setting of care. Furthermore, as prolonged in-hospital stay is known to be linked to raise in incidence of complications and hospital-acquired-infections, patients’ stay gets even more prolonged as planned discharge gets more and more postponed, with raise in healthcare costs, and diminishing benefit for the patient who is needlessly put at increased health risk [80].

### Generalizability

This work is a single-center observational study focused on a limited number of wards, whose common denominator is given by the fact that they mostly admit their patients from the same population, and with non-surgical necessities. While the organization of SSP hospital within ASL Roma 1 may be peculiar, its difficulties in the discharge process are not, as they are worldwide encountered [22, 81, 82]. A recent work of Manning and Islam highlighted that most of the challenges in patient flow management are not directly related to clinical situation, and solution must be found at organizational level [83]. To our knowledge, this is one of the few works that gives a magnitude of the Delayed Discharge phenomenon with a patient-level analysis. Other papers focused on estimation using excessive length of stay as a proxy, or focused on risk factors and causes from individual perspective [4, 76, 84]. This work adds evidence to the notion that delay in discharge process is not due to clinical factors but organizational ones. Such organizational elements transcend the hospital system as they represent the addition of a further level of uncertainty in a system that is structurally characterized by high levels of complexity. As such, solution and policies to the Delayed Discharge problems cannot be found exclusively inside the hospital system, but must encompass the entire galaxy of systems and facilities that revolves around hospital structures [31]. Quantification of direct and indirect costs of delayed discharge may help both policy makers and middle managers in deciding the most optimal way for resource allocation when designing their local health systems, in the ever-ongoing quest for providing each patient the right setting of care.

## Supporting information

S1 Checklist *PLOS ONE* clinical studies checklist. (DOCX)

S2 Checklist The RECORD statement–checklist of items, extended from the STROBE statement, that should be reported in observational studies using routinely collected health data. (DOCX)
